# 5,6-Dichloro-2-(3-methoxy­phen­yl)isoindoline-1,3-dione

**DOI:** 10.1107/S1600536808010659

**Published:** 2008-04-23

**Authors:** Orhan Büyükgüngör, Mustafa Odabaşoğlu

**Affiliations:** a Department of Physics, Faculty of Arts and Sciences, Ondokuz Mayıs University, TR-55139 Kurupelit Samsun, Turkey; b Department of Chemistry, Faculty of Arts and Sciences, Ondokuz Mayıs University, TR-55139 Kurupelit Samsun, Turkey

## Abstract

The title compound, C_15_H_9_Cl_2_NO_3_, crystallizes as an inversion twin, the ratio of the twin components being 0.43 (13):0.57 (13). The isoindoline group is planar and inclined by 77.63 (3)° to the aromatic ring substituent. The crystal structure is stabilized by aromatic π–π stacking inter­actions involving the benzene rings of adjacent isoindoline groups, with a centroid–centroid distance of 3.664 (7) Å and an inter­planar separation of 3.409 Å.

## Related literature

For general background, see: Chapman *et al.* (1979 ▶); Hall *et al.*, (1983 ▶; 1987 ▶); Srivastava *et al.* (2001 ▶); Abdel-Hafez (2004 ▶); Sena *et al.* (2007 ▶).
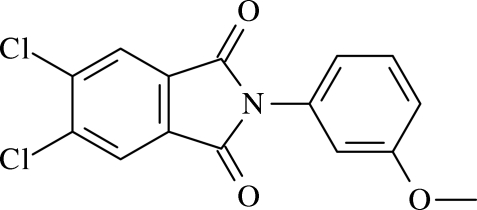


## Experimental

### 

#### Crystal data

C_15_H_9_Cl_2_NO_3_
*M* *_r_* = 322.13
Orthorhombic, 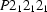

*a* = 6.8689 (5) Å
*b* = 9.7362 (10) Å
*c* = 20.4271 (14) Å
*V* = 1366.1 (2) Å^3^
*Z* = 4
Mo *K*α radiation
μ = 0.48 mm^−1^
*T* = 296 K
0.65 × 0.51 × 0.23 mm

#### Data collection

Stoe IPDSII diffractometer
Absorption correction: integration (*X-RED32*; Stoe & Cie, 2002 ▶) *T*_min_ = 0.685, *T*_max_ = 0.888
6123 measured reflections
2687 independent reflections
2076 reflections with *I* > 2σ(*I*)
*R*_int_ = 0.117

#### Refinement

*R*[*F* ^2^ > 2σ(*F* ^2^)] = 0.071
*wR*(*F* ^2^) = 0.169
*S* = 1.00
2687 reflections
192 parameters
H-atom parameters constrained
Δρ_max_ = 0.63 e Å^−3^
Δρ_min_ = −0.57 e Å^−3^
Absolute structure: Flack (1983 ▶), 1114 Friedel pairs
Flack parameter: 0.43 (13)

### 

Data collection: *X-AREA* (Stoe & Cie, 2002 ▶); cell refinement: *X-AREA*; data reduction: *X-RED32* (Stoe & Cie, 2002 ▶); program(s) used to solve structure: *SHELXS97* (Sheldrick, 2008 ▶); program(s) used to refine structure: *SHELXL97* (Sheldrick, 2008 ▶); molecular graphics: *ORTEP-3 for Windows* (Farrugia, 1997 ▶); software used to prepare material for publication: *WinGX* (Farrugia, 1999 ▶).

## Supplementary Material

Crystal structure: contains datablocks I, global. DOI: 10.1107/S1600536808010659/rz2205sup1.cif

Structure factors: contains datablocks I. DOI: 10.1107/S1600536808010659/rz2205Isup2.hkl

Additional supplementary materials:  crystallographic information; 3D view; checkCIF report

## Figures and Tables

**Fig. 1.**
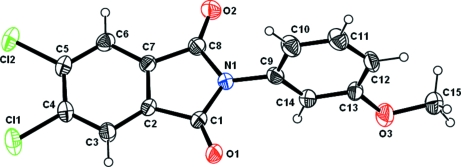
The molecular structure of the title molecule, with the atom-numbering scheme. Displacement ellipsoids are drawn at the 30% probability level.

**Fig. 2.**
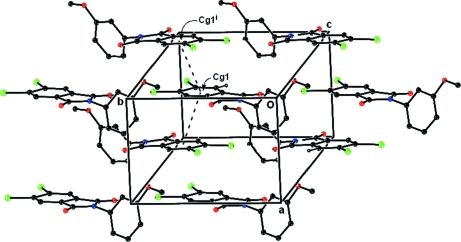
A partial packing diagram of the title compound, showing the formation of π···π stacking interactions. H atoms are omitted for clarity. Cg1 and Cg1i are the centroids of the benzene rings of the isoindoline groups. [Symmetry code: (i) -1/2+x, 3/2-y, 1-z].

## supplementary crystallographic information

### Comment

The present work is part of a structural study of derivatives of *N*-arylphthalimides (Abdel-Hafez, 2004; Chapman *et al.*, 1979; Hall *et al.*,  1983; Hall *et al.*,  1987; Sena *et al.*,  2007; Srivastava *et al.*,  2001). We report here the crystal structure of 5,6-dichloro-2-(3-methoxyphenyl)isoindoline-1,3-dione.

The molecule of the title compound consist of a 5,6-dichlorophthalimide unit connected to a *m*-methoxyphenyl group through the nitrogen atom (Fig. 1). The isoindoline ring (atoms N1/C1–C8) is almost planar, the largest deviation from the mean plane being 0.051 (3) Å for atom C5. The dihedral angle between the methoxyphenyl ring and the mean plane of the isoindoline group is 77.63 (3)°. The crystal packing is stabilized by aromatic π···π stacking interactions (Fig. 2) occurring between the aromatic rings of isoindoline groups at (*x*, *y*, *z*) and (-1/2+*x*; 3/2 - *y*; 1-*z*), with a centroid-centroid distance of 3.664 (7) Å and a plane-plane separations of 3.409 Å.

### Experimental

A mixture of 4,5-dichlorophthalic acid (1.175 g, 0.005 mol) and 2-aminophenol (0.545 g, 0.02 mol) in DMF (1.5 ml) was heated at boiling temperature for 15 min, then ethanol (95%, 50 ml) was added. Crystals of the title compound suitable for X-ray analysis were obtained by slow evaporation of this mixture at room temperature (yield 80%; mp. 546–548 K).

### Refinement

H atoms were positioned geometrically, with C—H = 0.93 and 0.96 Å for aromatic and methyl H, respectively, and constrained to ride on their parent atoms with *U*_iso_(H) = 1.2*U*_eq_(C) or 1.5*U*_eq_(C) for methyl H atoms. The rather high Rint value is ascribed to the poor nature of the available crystals.

### Crystal data

**Table:**

C_15_H_9_Cl_2_NO_3_	*F*_000_ = 656
*M**_r_* = 322.13	*D*_x_ = 1.566 Mg m^−^^3^
Orthorhombic, *P*2_1_2_1_2_1_	Mo *K*α radiation λ = 0.71073 Å
Hall symbol: P 2ac 2ab	Cell parameters from 6123 reflections
*a* = 6.8689 (5) Å	θ = 2.0–27.3º
*b* = 9.7362 (10) Å	µ = 0.48 mm^−^^1^
*c* = 20.4271 (14) Å	*T* = 296 K
*V* = 1366.1 (2) Å^3^	Prism, colourless
*Z* = 4	0.65 × 0.51 × 0.23 mm

### Data collection

**Table:**

Stoe IPDSII diffractometer	2687 independent reflections
Monochromator: plane graphite	2076 reflections with *I* > 2σ(*I*)
Detector resolution: 6.67 pixels mm^-1^	*R*_int_ = 0.117
*T* = 296 K	θ_max_ = 26.0º
ω scan rotation method	θ_min_ = 2.0º
Absorption correction: integration(X-RED32; Stoe & Cie, 2002)	*h* = −8→8
*T*_min_ = 0.685, *T*_max_ = 0.888	*k* = −12→10
6123 measured reflections	*l* = −23→25

### Refinement

**Table:**

Refinement on *F*^2^	H-atom parameters constrained
Least-squares matrix: full	*w* = 1/[σ^2^(*F*_o_^2^) + (0.1011*P*)^2^] where *P* = (*F*_o_^2^ + 2*F*_c_^2^)/3
*R*[*F*^2^ > 2σ(*F*^2^)] = 0.071	(Δ/σ)_max_ = 0.001
*wR*(*F*^2^) = 0.169	Δρ_max_ = 0.63 e Å^−^^3^
*S* = 1.01	Δρ_min_ = −0.57 e Å^−^^3^
2687 reflections	Extinction correction: SHELXL97 (Sheldrick, 2008), Fc^*^=kFc[1+0.001xFc^2^λ^3^/sin(2θ)]^-1/4^
192 parameters	Extinction coefficient: 0.054 (7)
Primary atom site location: structure-invariant direct methods	Absolute structure: Flack (1983), 1114 Friedel pairs
Secondary atom site location: difference Fourier map	Flack parameter: 0.43 (13)
Hydrogen site location: inferred from neighbouring sites	

### Special details

**Table:**

Geometry. All e.s.d.'s (except the e.s.d. in the dihedral angle between two l.s. planes) are estimated using the full covariance matrix. The cell e.s.d.'s are taken into account individually in the estimation of e.s.d.'s in distances, angles and torsion angles; correlations between e.s.d.'s in cell parameters are only used when they are defined by crystal symmetry. An approximate (isotropic) treatment of cell e.s.d.'s is used for estimating e.s.d.'s involving l.s. planes.
Refinement. Refinement of *F*^2^ against ALL reflections. The weighted *R*-factor *wR* and goodness of fit *S* are based on *F*^2^, conventional *R*-factors *R* are based on *F*, with *F* set to zero for negative *F*^2^. The threshold expression of *F*^2^ > σ(*F*^2^) is used only for calculating *R*-factors(gt) *etc*. and is not relevant to the choice of reflections for refinement. *R*-factors based on *F*^2^ are statistically about twice as large as those based on *F*, and *R*- factors based on ALL data will be even larger.

### Fractional atomic coordinates and isotropic or equivalent isotropic displacement parameters (Å^2^)

**Table:**

	*x*	*y*	*z*	*U*_iso_*/*U*_eq_	
C1	0.2767 (6)	0.3976 (4)	0.4834 (2)	0.0408 (9)	
C2	0.2607 (6)	0.5458 (4)	0.5011 (2)	0.0380 (8)	
C3	0.2552 (7)	0.6055 (4)	0.5626 (2)	0.0474 (9)	
H3	0.2551	0.5526	0.6005	0.057*	
C4	0.2499 (6)	0.7459 (4)	0.5652 (2)	0.0458 (9)	
C5	0.2520 (6)	0.8278 (4)	0.5080 (2)	0.0444 (9)	
C6	0.2545 (7)	0.7642 (4)	0.4471 (2)	0.0463 (9)	
H6	0.2538	0.8157	0.4088	0.056*	
C7	0.2579 (6)	0.6225 (4)	0.44509 (19)	0.0395 (8)	
C8	0.2677 (6)	0.5285 (4)	0.3883 (2)	0.0438 (9)	
C9	0.3137 (6)	0.2765 (5)	0.3758 (2)	0.0436 (10)	
C10	0.4978 (6)	0.2535 (6)	0.3505 (3)	0.0598 (13)	
H10	0.5993	0.3135	0.3599	0.072*	
C11	0.5282 (7)	0.1425 (6)	0.3118 (3)	0.0624 (14)	
H11	0.6517	0.1277	0.2946	0.075*	
C12	0.3806 (7)	0.0500 (5)	0.2972 (2)	0.0527 (12)	
H12	0.4041	−0.0263	0.2710	0.063*	
C13	0.1967 (7)	0.0752 (5)	0.3229 (2)	0.0477 (11)	
C14	0.1630 (6)	0.1861 (4)	0.3621 (2)	0.0466 (10)	
H14	0.0397	0.2011	0.3795	0.056*	
C15	0.0552 (10)	−0.1131 (6)	0.2632 (3)	0.0743 (16)	
H15A	−0.0652	−0.1629	0.2605	0.112*	
H15B	0.1584	−0.1749	0.2749	0.112*	
H15C	0.0834	−0.0720	0.2216	0.112*	
N1	0.2788 (5)	0.3963 (4)	0.41436 (16)	0.0412 (8)	
O1	0.2907 (5)	0.2994 (3)	0.51795 (15)	0.0550 (8)	
O2	0.2669 (6)	0.5562 (3)	0.33082 (15)	0.0616 (9)	
O3	0.0389 (5)	−0.0085 (4)	0.3116 (2)	0.0749 (11)	
Cl1	0.2389 (2)	0.82582 (13)	0.64049 (6)	0.0730 (4)	
Cl2	0.25735 (16)	1.00342 (11)	0.51245 (6)	0.0578 (4)	

### Atomic displacement parameters (Å^2^)

**Table:**

	*U*^11^	*U*^22^	*U*^33^	*U*^12^	*U*^13^	*U*^23^
C1	0.047 (2)	0.0320 (19)	0.044 (2)	−0.0019 (18)	0.0005 (19)	0.0014 (16)
C2	0.0407 (16)	0.0300 (17)	0.043 (2)	−0.0036 (16)	−0.0006 (19)	−0.0017 (14)
C3	0.058 (2)	0.041 (2)	0.043 (2)	0.004 (2)	−0.004 (2)	−0.0045 (17)
C4	0.0455 (19)	0.044 (2)	0.047 (2)	0.003 (2)	−0.006 (2)	−0.0117 (17)
C5	0.0383 (15)	0.0338 (19)	0.061 (2)	0.0010 (17)	−0.006 (2)	−0.0052 (18)
C6	0.0487 (19)	0.037 (2)	0.053 (2)	0.006 (2)	0.001 (2)	0.0073 (17)
C7	0.0399 (17)	0.0360 (19)	0.043 (2)	−0.0027 (18)	0.001 (2)	0.0000 (15)
C8	0.049 (2)	0.041 (2)	0.042 (2)	0.004 (2)	0.0003 (18)	0.0012 (16)
C9	0.054 (2)	0.043 (2)	0.034 (2)	0.0034 (19)	0.0016 (15)	0.0006 (18)
C10	0.050 (2)	0.072 (3)	0.058 (3)	−0.006 (2)	0.007 (2)	−0.016 (3)
C11	0.057 (3)	0.069 (4)	0.061 (3)	0.007 (3)	0.010 (2)	−0.010 (3)
C12	0.075 (3)	0.043 (3)	0.040 (3)	0.012 (2)	0.002 (2)	−0.011 (2)
C13	0.066 (3)	0.035 (2)	0.042 (2)	−0.0022 (19)	−0.0012 (18)	−0.0046 (18)
C14	0.054 (2)	0.040 (2)	0.046 (2)	0.0013 (19)	0.0058 (18)	−0.001 (2)
C15	0.108 (4)	0.059 (3)	0.055 (3)	−0.021 (3)	−0.001 (3)	−0.013 (3)
N1	0.0523 (19)	0.0362 (17)	0.0351 (18)	−0.0028 (15)	0.0025 (15)	−0.0036 (13)
O1	0.087 (2)	0.0336 (15)	0.0445 (17)	0.0030 (16)	−0.0010 (15)	0.0009 (12)
O2	0.090 (2)	0.0525 (18)	0.0425 (18)	0.004 (2)	0.0015 (18)	0.0047 (14)
O3	0.081 (2)	0.067 (2)	0.076 (3)	−0.026 (2)	0.0145 (19)	−0.027 (2)
Cl1	0.1007 (9)	0.0574 (7)	0.0609 (8)	0.0176 (8)	−0.0121 (8)	−0.0238 (6)
Cl2	0.0536 (5)	0.0314 (5)	0.0883 (8)	0.0032 (5)	−0.0054 (6)	−0.0070 (5)

### Geometric parameters (Å, °)

**Table:**

C1—O1	1.192 (5)	C9—C10	1.384 (6)
C1—N1	1.410 (5)	C9—C14	1.387 (6)
C1—C2	1.491 (5)	C9—N1	1.428 (5)
C2—C7	1.367 (5)	C10—C11	1.355 (7)
C2—C3	1.384 (6)	C10—H10	0.9300
C3—C4	1.368 (6)	C11—C12	1.388 (7)
C3—H3	0.9300	C11—H11	0.9300
C4—C5	1.415 (6)	C12—C13	1.390 (6)
C4—Cl1	1.725 (4)	C12—H12	0.9300
C5—C6	1.389 (6)	C13—C14	1.364 (6)
C5—Cl2	1.713 (4)	C13—O3	1.376 (5)
C6—C7	1.381 (6)	C14—H14	0.9300
C6—H6	0.9300	C15—O3	1.424 (6)
C7—C8	1.479 (6)	C15—H15A	0.9600
C8—O2	1.205 (5)	C15—H15B	0.9600
C8—N1	1.395 (5)	C15—H15C	0.9600
			
O1—C1—N1	125.7 (4)	C14—C9—N1	120.2 (3)
O1—C1—C2	129.6 (4)	C11—C10—C9	119.2 (4)
N1—C1—C2	104.6 (3)	C11—C10—H10	120.4
C7—C2—C3	122.0 (4)	C9—C10—H10	120.4
C7—C2—C1	109.0 (3)	C10—C11—C12	122.0 (4)
C3—C2—C1	129.0 (4)	C10—C11—H11	119.0
C4—C3—C2	117.1 (4)	C12—C11—H11	119.0
C4—C3—H3	121.5	C11—C12—C13	117.9 (4)
C2—C3—H3	121.5	C11—C12—H12	121.1
C3—C4—C5	122.0 (4)	C13—C12—H12	121.1
C3—C4—Cl1	119.1 (3)	C14—C13—O3	115.7 (4)
C5—C4—Cl1	118.9 (3)	C14—C13—C12	121.1 (4)
C6—C5—C4	119.2 (4)	O3—C13—C12	123.2 (4)
C6—C5—Cl2	119.5 (3)	C13—C14—C9	119.6 (4)
C4—C5—Cl2	121.2 (3)	C13—C14—H14	120.2
C7—C6—C5	118.2 (4)	C9—C14—H14	120.2
C7—C6—H6	120.9	O3—C15—H15A	109.5
C5—C6—H6	120.9	O3—C15—H15B	109.5
C2—C7—C6	121.4 (4)	H15A—C15—H15B	109.5
C2—C7—C8	108.6 (3)	O3—C15—H15C	109.5
C6—C7—C8	130.0 (4)	H15A—C15—H15C	109.5
O2—C8—N1	125.4 (4)	H15B—C15—H15C	109.5
O2—C8—C7	128.7 (4)	C8—N1—C1	111.9 (3)
N1—C8—C7	105.9 (3)	C8—N1—C9	123.5 (3)
C10—C9—C14	120.3 (4)	C1—N1—C9	124.1 (3)
C10—C9—N1	119.5 (4)	C13—O3—C15	118.6 (4)
			
O1—C1—C2—C7	176.9 (5)	C14—C9—C10—C11	0.5 (8)
N1—C1—C2—C7	−1.1 (5)	N1—C9—C10—C11	−177.4 (4)
O1—C1—C2—C3	−1.1 (8)	C9—C10—C11—C12	−0.6 (9)
N1—C1—C2—C3	−179.1 (4)	C10—C11—C12—C13	0.8 (8)
C7—C2—C3—C4	−1.0 (7)	C11—C12—C13—C14	−0.9 (7)
C1—C2—C3—C4	176.7 (4)	C11—C12—C13—O3	179.7 (5)
C2—C3—C4—C5	−0.6 (8)	O3—C13—C14—C9	−179.7 (4)
C2—C3—C4—Cl1	178.9 (3)	C12—C13—C14—C9	0.9 (7)
C3—C4—C5—C6	1.7 (7)	C10—C9—C14—C13	−0.7 (7)
Cl1—C4—C5—C6	−177.9 (4)	N1—C9—C14—C13	177.2 (4)
C3—C4—C5—Cl2	−176.8 (4)	O2—C8—N1—C1	180.0 (5)
Cl1—C4—C5—Cl2	3.6 (5)	C7—C8—N1—C1	0.1 (5)
C4—C5—C6—C7	−1.0 (7)	O2—C8—N1—C9	7.6 (7)
Cl2—C5—C6—C7	177.4 (3)	C7—C8—N1—C9	−172.3 (4)
C3—C2—C7—C6	1.7 (7)	O1—C1—N1—C8	−177.5 (4)
C1—C2—C7—C6	−176.5 (4)	C2—C1—N1—C8	0.6 (5)
C3—C2—C7—C8	179.3 (4)	O1—C1—N1—C9	−5.2 (7)
C1—C2—C7—C8	1.2 (5)	C2—C1—N1—C9	172.9 (3)
C5—C6—C7—C2	−0.6 (7)	C10—C9—N1—C8	72.8 (6)
C5—C6—C7—C8	−177.7 (4)	C14—C9—N1—C8	−105.2 (5)
C2—C7—C8—O2	179.3 (5)	C10—C9—N1—C1	−98.6 (5)
C6—C7—C8—O2	−3.3 (9)	C14—C9—N1—C1	83.4 (5)
C2—C7—C8—N1	−0.8 (5)	C14—C13—O3—C15	170.2 (5)
C6—C7—C8—N1	176.6 (5)	C12—C13—O3—C15	−10.5 (8)
